# Interpersonal Emotion Regulation Effort in Romantic Couples Across Adulthood

**DOI:** 10.1093/geroni/igaf122.1396

**Published:** 2025-12-31

**Authors:** Tammy English, Claire Growney, Jocelyn Lai, Junyuan Luo

**Affiliations:** Washington University in St. Louis, St. Louis, Missouri, United States; Stanford University, Stanford, California, United States; Washington University in St. Louis, St. Louis, Missouri, United States; Washington University in St. Louis, St. Louis, Missouri, United States

## Abstract

Extrinsic interpersonal emotion regulation (IER) refers to when an individual (the regulator) attempts to regulate the emotions of another person (the target). Outcomes of IER are theorized to depend on both the strategy one deploys and the regulator’s motivational strength or effort put into IER. Greater effort is expected to lead to greater success in helping the target resolve the stressor and may strengthen the regulator-target relationship. However, there may be personal costs to the regulator due to the demands of IER. Given age-related shifts in motivational priorities and accumulated life experience, we expected IER effort to be more effective and less costly for older adults. To test these ideas, we recruited romantic couples (Study 1 = 134, Study 2 = 121) across the adult lifespan (age 23-92) to complete 9 days of daily diaries and engage in an emotional conversation in the lab. Using actor-partner interdependence modeling, we found that greater IER effort was generally associated with more positive emotional and relational outcomes in both partners. However, IER effort was also linked to greater negative emotion in the regulator, especially when this support was under-acknowledged. IER was more strongly linked to wellbeing outcomes for relatively older targets. Results contribute to a deeper understanding of the motivational aspect of emotion regulation across adulthood. Putting in greater effort to manage the emotions of a close partner may have mixed consequences for both younger and older adults.

